# T cell intrinsic STAT1 signaling prevents aberrant Th1 responses during acute toxoplasmosis

**DOI:** 10.3389/fimmu.2023.1212190

**Published:** 2023-07-25

**Authors:** Aaron B. Schultz, David G. Kugler, Luis Nivelo, Nicolas Vitari, Laura P. Doyle, Svetlana Ristin, Lothar Hennighausen, John J. O’Shea, Dragana Jankovic, Alejandro V. Villarino

**Affiliations:** ^1^ Department of Microbiology and Immunology, Miller School of Medicine, University of Miami, Miami, FL, United States; ^2^ Sylvester Comprehensive Cancer Center, University of Miami, Miami, FL, United States; ^3^ Immunoparasitology Unit, Laboratory of Parasitic Diseases, National Institute of Allergy and Infectious Diseases, National Institutes of Health, Bethesda, MD, United States; ^4^ Department of Surgery, Miller School of Medicine, University of Miami, Miami, FL, United States; ^5^ Department of Molecular and Cellular Pharmacology, Miller School of Medicine, University of Miami, Miami, FL, United States; ^6^ National Institute of Diabetes, Digestive and Kidney Diseases, National Institutes of Health, Bethesda, MD, United States; ^7^ Lymphocyte Cell Biology Section, National Institute of Arthritis, Musculoskeletal and Skin Diseases, National Institutes of Health, Bethesda, MD, United States

**Keywords:** JAK - STAT signaling pathway, cytokine, STAT1, STAT3, IL-27 (interleukin 27), IL-10 (interleukin 10), toxoplasama gondii, IL-13 (interleukin 13)

## Abstract

Infection-induced T cell responses must be properly tempered and terminated to prevent immuno-pathology. Using transgenic mice, we demonstrate that T cell intrinsic STAT1 signaling is required to curb inflammation during acute infection with *Toxoplasma gondii*. Specifically, we report that mice lacking STAT1 selectively in T cells expel parasites but ultimately succumb to lethal immuno-pathology characterized by aberrant Th1-type responses with reduced IL-10 and increased IL-13 production. We also find that, unlike STAT1, STAT3 is not required for induction of IL-10 or suppression of IL-13 during acute toxoplasmosis. Each of these findings was confirmed *in vitro* and ChIP-seq data mining showed that STAT1 and STAT3 co-localize at the *Il10* locus, as well as loci encoding other transcription factors that regulate IL-10 production, most notably *Maf* and *Irf4*. These data advance basic understanding of how infection-induced T cell responses are managed to prevent immuno-pathology and provide specific insights on the anti-inflammatory properties of STAT1, highlighting its role in shaping the character of Th1-type responses.

## Introduction

*Toxoplasma gondii* is an obligate intracellular parasite that can elicit immuno-pathology (1, 2). IL-10 deficient mice (*Il10* ^-/-^) offer a striking example in that they efficiently control parasite replication but ultimately succumb to lethal T cell hyperactivity (3). Mice lacking IL-27 receptor (*Il27ra^-/-^*) bear a similar phenotype and it is now understood that IL-27R signaling is critical for T cell IL10 production which, in turn, is critical for limiting pathogenic T cell responses during infection (4–8). IL-27 can induce IL-10 production from multiple CD4^+^ T cell subsets, including FoxP3 ^pos^ T regulatory (Treg), FoxP3 ^neg^ Tr1, Th1 and Th17 cells (7–14). IFN-γ-producing Th1 cells are the main source during acute toxoplasmosis, so they are the focus here (15). Crucially, IL-10 production enables Th1 cells to at once encourage and suppress inflammation, thereby ensuring that it is robust enough to kill parasites yet tempered to prevent tissue damage (16). Both the IL-27 receptor (IL-27R) and IL-10 receptor (IL-10R) mobilize the JAK-STAT signaling pathway, with STAT1 and STAT3 viewed as key downstream mediators. Prior work has shown that STAT1 and STAT3 are each required for IL-27-driven IL-10 production *in vitro* (8, 17), but whether they are required *in vivo* remains to be determined. Prior work has also showed that STAT3 hyperactivity can suppress protective Th1 responses following infection with *T. gondii* but, again, the impact of STAT3 loss-of-function remains unclear (18).

Due to its central role in anti-viral and anti-microbial pathways, STAT1 is generally viewed as a pro-inflammatory transcription factor (19). Accordingly, STAT1 has multiple downstream functions that encourage protective Th1 responses during infection with *T. gondii*. Chief among these is its ability to induce expression of T-bet (*Tbx21*), a transcription factor that underlies key aspects of the Th1 differentiation program (20–23). Crucially, IL-27 is a potent T-bet inducer in CD4^+^ T cells and does so mainly *via* STAT1, which localizes to the *Tbx21* (T-bet) locus and thereby instructs transcription (17, 24–26). It is also important to note that, unlike *Il27ra^-/-^* mice, *Stat1^-/-^* and *Tbx21^-/-^* mice are each unable to control *T. gondii* replication, attendant to defective Th1 responses (27, 28). STAT1 also has important anti-inflammatory functions that are relevant for toxoplasmosis. Notable among these is its ability to limit Th2- and Th17-type responses (29–34), which it achieves, in part, by localizing to and modulating expression of key genes involved with these subsets, including cytokines IL-4, IL-13, IL-17A, IL-17F and IL-22, and transcription factors Gata-3, RORα and RORγt (17). It also influences Treg, a CD4^+^ T cell subset that is specialized to curb immunopathology (35). Specifically, IL-27-driven STAT1 signaling induces expression of T-bet in Treg, thereby ensuring that they co-localize with Th1 and Tc1 cells due to analogous, T-bet-driven changes in chemokines, chemokine receptors and adhesion molecules (9–11). Thus, STAT1 has both pro- and anti-inflammatory functions that are critical for mounting effective T cell responses against *T. gondii*.

Using a genetic approach, we demonstrate that mice lacking STAT1 specifically in T cells are able to control *T. gondii* replication but ultimately succumb to lethal immune-pathology characterized by aberrant Th1 cells with conspicuously reduced IL-10 and elevated IL-13 production. Importantly, ChIP-seq data mining also showed that, downstream of interleukin-27, STAT1 localizes to both the *Il10* and *Il13* loci downstream of interleukin-27, and to loci encoding transcription factors known to regulate IL-10 production, most notably *Maf* and *Irf4*. Conversely, STAT3 was dispensable for both promoting IL-10 and suppressing IL-13 production, despite the fact that it often co-localizes with STAT1 at these and other relevant gene loci (e.g. *Maf*, *Irf4*). Taken together, our data connect the dots between IL-27R and IL-10 in the context of *T. gondii* infection and advance the idea that STAT1 signaling endows Th1 cells with anti-inflammatory properties necessary to prevent immuno-pathology.

## Results

### T cell intrinsic STAT1 signaling is required for resistance to *T. gondii*

To study T cell intrinsic STAT1 signaling, we generated *Stat1 ^T-KO^* mice which lack STAT1 selectively in CD4^+^ and CD8^+^ T cells starting at the double-positive stage of thymic development. Prior to infection, total CD4^+^ and CD8^+^ T cells counts were comparable to WT littermates, although, consistent with studies in *Stat1^-/-^* mice (36), effector and memory cells were increased, as were FOXP3^+^ Treg (**Figures S1A–G**). Crucially, baseline frequencies of IFN-γ producing CD4^+^ and CD8^+^ T cells were also comparable to WT controls (**Figure S1I**).

**Figure 1:**
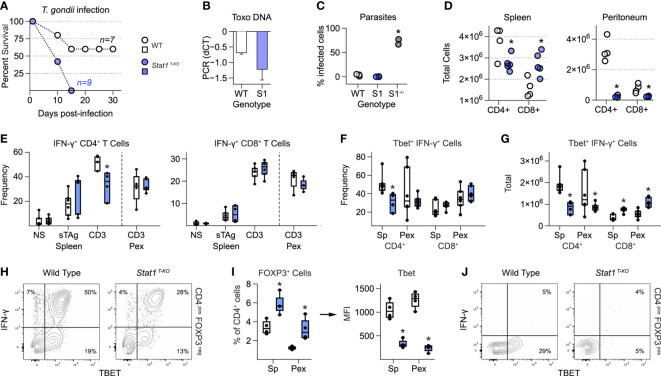
T cell intrinsic STAT1 signaling limits immuno-pathology in mice challenged with T. gondii. **(A)** Kaplan–Meier plot shows survival of infected WT and STAT1 ^T-KO^ mice. **(B)** Bar plot shows amplification of *T. gondii* DNA in livers at day 7 post-infection. Negative ΔΔCt values indicate that parasite DNA is less abundant than mammalian DNA. **(C)** Scatter plot shows parasite counts from peritoneal exudates (PEx) at day 7. S1 = *Stat1 ^T-KO^*; S1^-/-^ = *Stat1^-/-^* (pan-cellular KO). **(D)** Scatter plots show T cell counts in spleen and PEx at day 7. **(E-H)** Single cell suspensions from spleens and PEx were cultured overnight with soluble Toxoplasma antigen (sTAg) or immobilized anti-CD3ϵ. **(E)** Box plots show percentages of IFN-γ^+^ cells within the CD4^+^ and CD8^+^ compartments at day 7. **(F, G)** Box plots show percentage **(F)** and total **(G)** T-bet^+^ IFN-γ^+^ cells at day 7. **(H)** Flow cytometry contour plots show T-bet versus IFN-γ in splenic CD4^+^ T cells stimulated with anti-CD3ϵ (day 7). **(I)** Left box plot show percentage of FoxP3^+^ CD4^+^ in spleen and PEx at day 7. Right box plot shows mean fluorescence intensity for T-bet within FOXP3^+^ Treg. **(I)** Flow cytometry contour plots show T-bet and IFN-γ in splenic CD4^+^ FoxP3^+^ T cells stimulated with anti-CD3ϵ (day 7). **(A–J)** Color scheme from **(A)** is valid for all panels. Data are pooled from 3 experiments, each with at least 2 mice per group. Stars denote unpaired t-test *p* values <0.05 relative to WT controls. All box plots contain at least 3 biological replicates/group and points denote individual replicates. **(H, J)** Data are representative of 4 separate experiments.

Strikingly, upon challenge with *T. gondii*, *Stat1 ^T-KO^* mice were able to control parasite replication but invariably succumbed 1-2 weeks post infection (**Figures 1A–C**). This was in stark contrast to WT controls, which typically survived to chronic disease (>1 month), and *Stat1^-/-^* mice, which had rampant parasites (**Figure 1C**). We also noted histological lesions in spleen, liver and lungs reminiscent of *Il10-/-* and *Il27ra-/-* mice infected with *T. gondii* (**Figure S2A**) (3, 4). Total splenic CD4^+^ and CD8^+^ T cells were respectively lower and higher in *Stat1 ^T-KO^* mice (**Figure 1D**), and both lineages were sharply reduced at the site of infection (i.e. peritoneum), likely reflecting efficient parasite clearance (**Figure 1D**). Consistent with this latter point, serum levels of IFN-γ and IL-12p40, key cytokines driving anti-Toxoplasma responses (1, 2, 37), were substantially higher in *Stat1 ^T-KO^* mice that in WT controls (**Figure S2B**). Taken together, these data establish that T cell intrinsic STAT1 signaling is required for resistance to *T. gondii* and point to anti-inflammatory properties as most relevant.

To further assess T cell responses, we made single cell suspensions from spleens and peritoneal exudates (PEx) at seven days post-infection, then measured IFN-γ production in response to soluble Toxoplasma Antigen (sTAg) or agonist anti-T cell receptor antibodies (anti-CD3ϵ). IFN-γ is produced by both CD4^+^ Th1 and CD8^+^ Tc1 effector T cells, and strictly required for resistance to *T. gondii* (2). It is also a key STAT1 stimulus and, like STAT1, known to have anti-inflammatory properties (38). Frequencies of IFN-γ^+^ CD4^+^ and IFN-γ^+^ CD8+ T cells were comparable between WT and *Stat1 ^T-KO^* mice at seven days post-infection with one notable exception: splenic CD4^+^ T cells re-stimulated with anti-CD3ϵ (**Figure 1E** and **Figure S3A**). However, it should also be noted that due to differences in overall cellularity (**Figure 1D**), there were always fewer total IFN-γ^+^ CD4^+^ and more total IFN-γ^+^ CD8^+^ present in S*tat1 ^T-KO^* cultures, regardless of re-stimulation conditions (**Figures S3B, C**). Nevertheless, it is clear that despite a key role *in vitro* and other *in vivo* settings (22, 29), T cell intrinsic STAT1 signaling is not strictly required for IFN-γ production during acute toxoplasmosis.

T-bet is a key driver of T cell interferon production and Th1 differentiation (21). Given that T-bet is directly induced by STAT1 (22, 23) and required for resistance to *T. gondii* (27), we quantified T-bet^+^ IFN-γ^+^ T cells at day 7 post-infection. This analysis revealed a clear difference between the CD4^+^ and CD8^+^ compartments; CD4^+^ T-bet^+^ Th1 cells were significantly reduced in S*tat1 ^T-KO^* mice but CD8^+^ T-bet^+^ Tc1 cells were not (**Figures 1F, G** and **Figure S3A**). Thus, we conclude that STAT1 is required for optimal Th1-type responses but dispensable for parallel Tc1-type responses. However, we also emphasize that T-bet^+^ IFN-γ^+^ CD4^+^ T cells were still plainly evident in S*tat1 ^T-KO^* mice (**Figure 1H**), suggesting that STAT1-independent mechanisms are also in play, as previously suggested (28).

STAT1 both inhibits *de novo* Treg differentiation (39) and induces expression of T-bet in mature Treg, thereby enhancing their capacity to suppress Th1- and Tc1-type responses (9, 40, 41). Both properties were evident in S*tat1 ^T-KO^* mice following infection with *T. gondii*. The overall frequency of CD4^+^ Foxp3^+^ Treg was increased relative to WT controls while, at the same time, the proportion of those expressing T-bet was sharply reduced (**Figures 1I, J**). Given that *T-bet^-/-^* Treg are defective in the context of *T. gondii* infection (42), we interpret that a lack of T-bet^+^ Treg likely contributes to emergence of pathogenic T cell responses in S*tat1 ^T-KO^* mice.

### STAT1 deficiency results in aberrant Th1 responses

**Figure 2:**
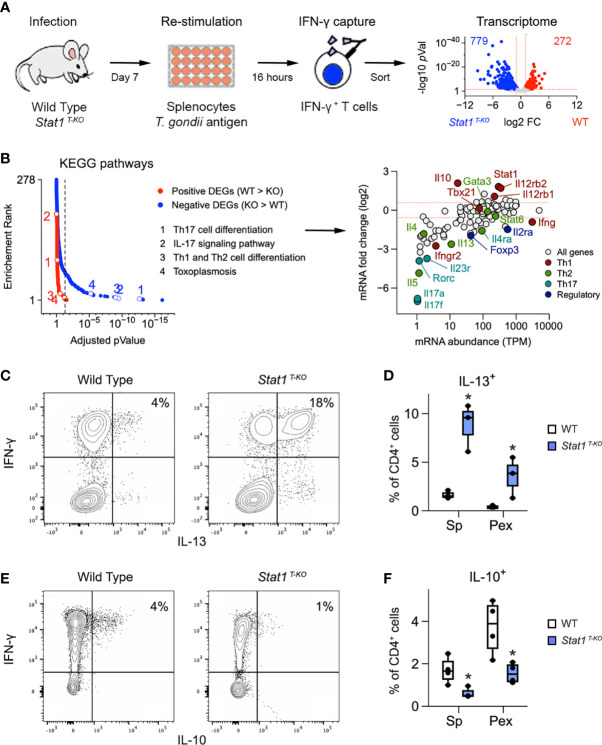
Aberrant Th1 responses in the absence of STAT1. **(A)** Cartoon shows experimental design for transcriptome studies. Volcano plot shows positive (red) and negative (blue) DEG. See **Figure S4A** for sort gating and **Table S1** for full DEG list. **(B)** Positive and negative DEG were tested for biological pathway enrichment against the KEGG database. Line graph shows *p* values and *p* value ranks for all intruded pathways. Select pathways are noted. Full dataset in **Table S2**. MA plot shows all genes from the KEGG Th1/Th2, Th17 and toxoplasmosis pathways. Select genes are called out and color coded based on the T cell subset that they are most associated with. **(C, E)** Flow cytometry contour plots show **(C)** IL-13 or **(E)** IL-10 versus IFN-γ in splenic CD4^+^ T cells stimulated with anti-CD3ϵ on day 7 post-infection. **(D, F)** Box plots show percentage of CD4^+^ single-positive **(D)** IL-13^+^ or **(E)** IL-10^+^ cells in spleen and PEx stimulated with antiCD3ϵ on day 7. **(A, B)** Data are from one experiment with 2 biological replicates per group. **(C, E)** Data are representative of 4 separate experiments. **(D, F)** Data are pooled from 3 experiments, each with at least 2 mice per group. Stars denote unpaired t-test *p* values <0.05 relative to WT controls. All box plots contain at least 3 biological replicates/group and points denote individual replicates.

Considering that *Stat1 ^T-KO^* mice generate robust Th1 and Tc1 responses when challenged with *T. gondii*, we reasoned that STAT1 deficiency may unleash pathogenic aspects IFN-γ-producing T cells. To engage this hypothesis, we sorted IFN-γ producing CD4^+^ T cells from infected WT and *Stat1 ^T-KO^* mice and compared transcriptomes by RNA-seq (**Figure 2A** and **Figure S4A**). Downstream analysis uncovered >1000 Differentially Expressed Genes (DEG), 74% of which were higher expressed in STAT1-deficient cells (**Figure 2A** and **Table S1).** Next, we tested positive and negative DEG for biological pathway enrichment against the KEGG database. Predictably, almost all enrichment was contained within the larger negative fraction (i.e. DEG that were higher expressed in *Stat1 ^T-KO^*), with T cell and Toxoplasma related pathways prominently represented (**Figures 2B**, **S4B, C** and **Table S2**).

The surprise came when we inspected the ‘hits’ within these pathways and found several genes involved with Th2- and Th17-type responses (**Figure 2B**). This was remarkable because Th2- and Th17-type cells are typically scarce during acute toxoplasmosis. Two Th2-related hits, *ll4ra* and *ll13*, were especially notable because they were substantially expressed (TMP >10; **Figure 2B**). The rest were far less abundant, typically < 5 TPM (**Figure 2B**). Flow cytometry confirmed that IFN-γ^+^ IL-13^+^ double-positive CD4^+^ T cells were readily detectable in S*tat1 ^T-KO^* mice at day 7 post-infection (**Figures 2C, D**, **S3C**), while IFN-γ^+^ IL-4^+^ and IFN-γ^+^ IL-17A^+^ double-positive cells were not (**Figures S3C–G**). However, while far less abundant than IL-13^+^ single-positive cells (**Figure S3C**), IL-4^+^ and IL-17A^+^ single-positive cells were still increased relative to WT controls (**Figures S3D–G**), consistent with prior work showing that STAT1 can suppress Th2- and Th17-type responses (29, 43, 44). Flow cytometry also confirmed that IL-13^+^ CD4^+^ T cells express high levels GATA3, the lineage-defining transcription factor for Th2 cells (**Figure S3H**). Transcript levels for *Il5*, another emblematic Th2 cytokine, and *Il17f*, another emblematic Th17 cytokine, were as low as *Il4*, suggesting that the former are also not evident at protein level (**Figure 2B** and **Figure S3D**). Thus, T cells from infected S*tat1 ^T-KO^* mice appear poised to express multiple Th2- and Th17-type cytokines but IL-13 is uniquely de-repressed and, given its pro-inflammatory nature, we propose that it contributes to the attendant immuno-pathology.

Aside from negative DEG, we also considered the possibility that positive DEG may contribute to the aberrant T cell responses seen in S*tat1 ^T-KO^* mice. We were immediately drawn to IL-10, which is required to suppress T cell responses during infection with *T. gondii*, as well as numerous other pathogens (16, 45). Strikingly, we found that IL-10 was dramatically reduced at both transcript and protein levels relative to WT controls (**Figures 2B, E, F**). We also noted that double-positive IFN-γ^+^ IL-10^+^ cells, the main IL-10 producers in WT mice, were scarce in S*tat1 ^T-KO^* mice (**Figure 2E**), and that IL-10-producing Treg cells were also diminished (**Figure S5**), suggesting that STAT1-driven IL-10 production may be relevant across T cell subsets. Consistent with this latter point, serum IL-10 was also diminished, indicting systemic failure of IL-10 production (**Figure S2B**). Given the many known anti-inflammatory properties of IL-10, we interpret that diminished production of this cytokine likely contributes to immune-pathology in *Stat1 ^T-KO^* mice challenged with *T. gondii*.

Along with anti-inflammatory features, our RNA-seq studies also illustrate pro-inflammatory features of STAT1 that are relevant during *T. gondii* infection. *Ifng* and *Tbx21* transcripts were each comparable to WT controls, likely due to the pre-selection of IFN-γ^+^ cells, but both IL-12 receptors subunits were sharply reduced in STAT1-deficient cells (**Figure 2B**). This is in line with prior work showing that STAT1 induces expression of *Il12rb1* and *Il12rb2* and notable here because IL-12 can include both IFN-γ and IL-10 production in T cells, and is strictly required for resistance to *T. gondii* (37). Taken together, our cytometry and RNA-seq studies affirm that T cell intrinsic STAT1 signaling has both pro-and anti-inflammatory functions which are critical for resistance to *T. gondii* and further argue that only the latter are nonredundant.

### STAT1 instructs IL-10 and IL-13 production in Th1 cells

**Figure 3:**
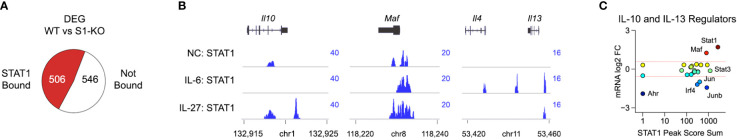
STAT1 directly engages Il10, Il13 and key upstream regulators. **(A)** DEG from **Figure 2A** were cross-referenced with STAT1 ChIP-seq data captured in CD4^+^ T cells cultured with IL-27. Pie chart shows proportion of DEG that are proximally bound by STAT1. **(B)** Genome browser tracks show STAT1 localization at the *Il10*, *Maf* and extended *Il13* loci. **(C)** Scatter plot shows cumulative STAT1 peak amplitude and transcript fold change (WT versus STAT1 ^T-KO^) for transcription factors known to regulate IL-10 and/or IL-13. Full listing in **Table S4**. Dotted red lines denote 2-fold changes.

STAT family transcription factors control gene expression in both direct and indirect ways (46). Direct regulation involves binding to DNA regulatory elements, most notably promoters and enhancers, and thereby instructing transcription of associated genes. Indirect regulation can take many forms but, here, we will focus on the ability of STATs to induce expression of other transcription factors which, in turn, mediate downstream effects. To address direct regulation, we cross-referenced DEG from our transcriptome studies with a STAT1 ChIP-seq dataset captured in CD4^+^ T cells cultured with IL-6 or IL-27 (**Table S3**) (17). This analysis revealed a near even split between STAT1 bound and ‘unbound’ DEG, indicating that both direct and indirect forms of regulation are relevant (**Figure 3A**). We were also struck by the appearance of both IL-10 and IL-13 among the STAT1 bound fraction as they have reciprocal expression patterns (i.e. positively versus negatively regulated) and likely play opposing roles during acute toxoplasmosis (i.e. anti- versus pro-inflammatory) (**Figure 2B** and **Figure 3B**).

To further explore their relationship to STAT1, we curated a list of transcription factors that are known to regulate IL-10 or IL-13 and asked which are proximally bound downstream of IL-27 (**Table S4**) (10, 45, 47–50). Surprisingly, we found that, while only a select few were called DEG in our transcriptome studies, nearly all were strongly bound by STAT1 (**Figure 3C** and **Figure S6**). Given the ambiguity of genes bound by STAT1 but not called DEG, which cannot be readily included or excluded as direct targets, we next focused on genes that met both criteria. MAF immediately stood out because it is both critical for T cell IL-10 production and strongly diminished in STAT1-deficient cells (**Figures 3B, C**). By contrast, *Irf4* was higher expressed in STAT1-deficient cells, as were AP-1 family transcription factors *Jun* and *Junb* (**Figures 3B, C** and **Figure S6**). Also notable, STAT1 itself was the most strongly bound IL-10 regulator (**Figure 3C**), in line with the long-standing notion that STATs induce themselves (46). Taken together, these data argue that STAT1 controls T cell IL-10 and IL-13 production through both direct and indirect mechanisms.

### Distinct roles for STAT1 and STAT3 in limiting aberrant Th1 responses

**Figure 4:**
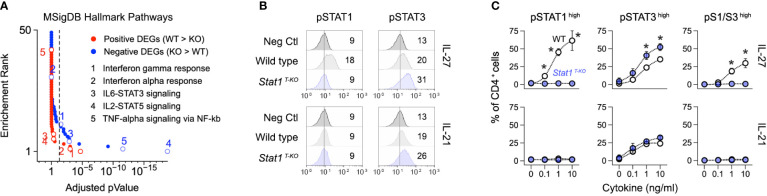
Enhanced STAT3 signaling in the absence of STAT1. **(A)** DEG from **Figure 2A** were tested against the Molecular Signatures Database. Line graph shows *p* values and p value ranks for all intruded pathways. Select pathways are noted. Full dataset in **Table S2**. **(B, C)** Naive CD4^+^ T cells were purified from uninfected WT and STAT1 ^T-KO^ mice, then activated and cultured in the presence of IL-27 or IL-21. **(B)** Flow cytometry histograms show modal renderings of STAT1 and STAT3 phosphorylation (p-Y701 and p-Y703, respectively). **(C)** Line graphs show percentages of cells with high p-STAT1 and/or pSTAT3 levels across the indicated cytokine concentrations. **(A)** Data are from one experiment with 2 biological replicates per group. **(B)** Data are representative of 5 separate experiments. Mean fluorescent intensity is noted. **(C)** Data are pooled from 3 experiments. Stars denote unpaired t-test *p* values <0.05 relative to WT controls (no cytokine).

We and others have shown that STAT3 signaling is enhanced in STAT1-deficient T cells, leading to enhanced STAT3-driven gene transcription (17, 51). Thus, we were intrigued to find that ‘STAT3 signaling’ was among the most enriched pathways in Th1 cells from infected S*tat1 ^T-KO^* mice (**Figure 4A**). Building on this finding, we confirmed that phosphorylation of STAT3 at tyrosine 705, the instigating event for canonical STAT3 signaling, is enhanced in STAT1-deficient T cells. Importantly, increased STAT3 phosphorylation was evident whether cells were cultured with IL-27, a potent activator of both STAT1 and STAT3, or IL-21, a potent STAT3 activator but lesser STAT1 activator (**Figures 4B, C**).

To further probe the relationship between STAT1 and STAT3, we crossed S*tat1 ^T-KO^* mice with *Stat3 ^HIES^* mice, which bear a germline transgene encoding a dominant negative form of STAT3 that impairs DNA binding (52). Of note, this mutation is commonly found in humans afflicted with Hyper IgE Syndrome (HIES), a disease characterized by severe immunological abnormalities, including massively elevated serum immunoglobulins impaired vaccination responses, and impaired Th17-type responses leading to recurrent fungal and bacterial infections (53). The resulting S*tat1 ^T-KO HIES^* mice therefore lack STAT1 selectively in T cells and have reduced STAT3 activity in all cells. They were born at expected mendelian rations and indistinguishable from WT littermates in terms of appearance and lifespan (not shown). This was in line with prior studies on the parent *Stat3 ^HIES^* strain (52) but in stark contrast to humans afflicted with HIES, most of whom exhibit severe developmental and skeletal defects (53). Notwithstanding, the progeny S*tat1 ^T-KO HIES^* strain mirrored the parent *Stat3 ^HIES^* strain in that both bore key immunological features of HIES, including prominent serum IgE (**Figure S2C**) and impaired Th17 responses (**Figures S7A, B**). They also mirrored the parent *Stat3 ^HIES^* strain in terms of increased CD8^+^ T cell IFN-γ production (**Figure S1I**), and, notably, far eclipsed them in terms of serum IgE levels (**Figure S2C**). Most of the other immunological features that were considered, including total and memory CD8^+^ T cells, more closely resembled the parent S*tat1 ^T-KO^* strain (**Figures S1D–F**).

**Figure 5:**
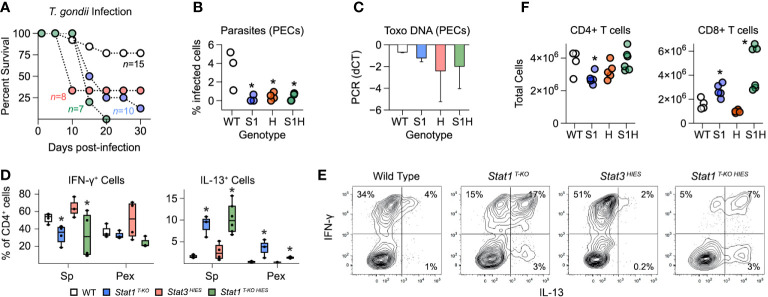
Distinct roles for STAT1 and STAT3 in limiting aberrant Th1 responses. **(A)** Kaplan–Meier plot shows survival of infected WT, STAT1 ^T-KO^, STAT3 ^HIES^ and STAT1 ^T-KO HIES^ mice. Genotype color scheme shown in **(D)**. **(B)** Scatter plot shows parasite counts from PEx at day 7 post-infection. **(C)** Bar plot shows PCR amplification of *T. gondii* DNA relative to mammalian DNA in livers at day 7. **(D, E)** Single cell suspensions from spleens and PEx were cultured overnight with immobilized anti-CD3ϵ. **(D)** Box plots show percentages of CD4^+^ single-positive IFN-γ ^+^ or IL-13^+^ cells within spleen and PEx at day 7. **(E)** Flow cytometry contour plots show IL-13 versus IFN-γ in splenic CD4^+^ T cells stimulated with anti-CD3ϵ on day 7. Data are representative of 4 separate experiments. **(F)** Scatter plots show CD4^+^ and CD8^+^ T cell counts in spleens at day 7. **(A-F)** Color scheme from **(D)** is valid for all panels. Data are pooled from 3 experiments, each with at least 2 mice per group. Stars denote unpaired t-test *p* values <0.05 relative to WT controls. All box plots contain at least 3 biological replicates/group and points denote individual replicates.

S*tat1 ^T-KO HIES^* mice largely phenocopied *Stat1 ^T-KO^* mice when challenged with *T. gondii*. They were able to control parasite replication but ultimately succumbed to immune-pathology characterized by aberrant, IL-13 producing Th1 cells (**Figures 5A–E**). *STAT3 ^HIES^* mice also cleared the parasites and succumbed to immunopathology but T cells from these animals produced substantially more IFN-γ than WT controls and did not produce IL-13 (**Figures 5A–E**). It was also notable that S*tat1 ^T-KO HIES^* mice varied from both parental strains in terms of total CD4^+^ and CD8^+^ T cell counts (**Figure 5F**). Nevertheless, we can conclude that STAT1 is uniquely required to limit outgrowth of IL-13-producing Th1 cells during acute toxoplasmosis.

**Figure 6:**
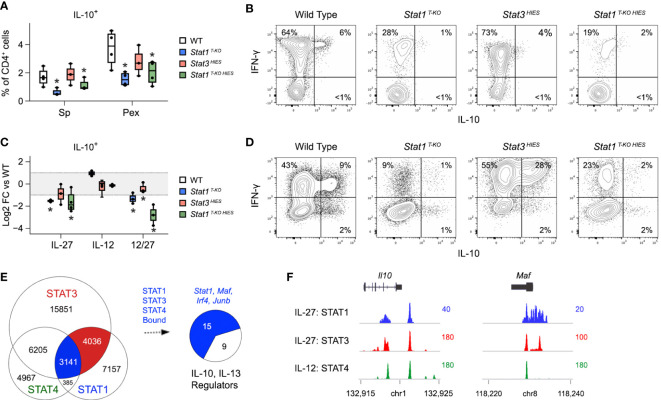
STAT1 is uniquely required for IL-10 production under Th1 polarizing conditions.**(A, B)** Single cell suspensions were made from spleens and PEx at day 7 post-infection and cultured overnight with immobilized anti-CD3ϵ. **(A)** Box plots show percentages of single-positive IL-10^+^ cells within the CD4^+^ compartment. Data are pooled from 3 experiments, each with at least 2 mice per group. **(B)** Flow cytometry contour plots show IL-10 versus IFN-γ in splenic CD4^+^ T cells. **(C, D)** Naive CD4^+^ T cells were purified from uninfected mice, then activated and cultured in the presence of IL-12 and/or IL-27 for 72 hours. **(C)** Box plots show log2 fold change in percentage of IL-10^+^ CD4^+^ cells relative to WT controls. Data are pooled from 3 experiments, each with 1 mouse per group. **(D)** Flow cytometry contour plots show IL-10 versus IFN-γ in CD4^+^ cells cultured with IL-12 and IL-27. **(E)** STAT1, STAT3 and STAT4 ChIP-seq datasets were cross-referenced. Venn plot enumerates overlapping genomic regions. Blue union denotes regions bound by all 3 STATs. Pie chart shows proportion of IL10 and IL-13 regulators (from **Figure 3C**) that are proximally bound by STAT1, STAT3 and STAT4. Full listing in **Table S4**. **(F)** Genome browser tracks show STAT co-localization at the *Il10* and *Maf* loci. **(A–D)** Stars denote unpaired t-test *p* values <0.05 relative to WT controls. All box plots contain at least 3 biological replicates/group and points denote individual replicates.

STAT1 and STAT3 have each been implicated in the generation of IL-10-producing T cells during *T. gondii* infection (8). However, despite strong *in vitro* evidence, neither has been shown to be strictly required *in vivo*. Thus, we were intrigued to find that CD4^+^ IFN-γ^+^ IL-10^+^ T cells were nearly absent in *Stat1 ^T-KO HIES^* mice but not STAT3 *^HIES^* mice, which were comparable to WT controls (**Figures 6A, B**). These data formally establish that STAT1 is dominant *in vivo*, so we next compared IL-10 production *in vitro* under conditions that mimic the inflammatory milieu of acute toxoplasmosis. Specifically, naive CD4^+^ T cells were cultured in the presence of IL-12 and IL-27, then cytokine production assayed by flow cytometry. Again, we found that the frequency of double-positive IFN-γ^+^ IL-10^+^ and single-positive IL-10^+^ events was far lower in S*tat1 ^T-KO^* and *Stat1 ^T-KO HIES^* cultures than in *STAT3 ^HIES^* cultures, indicating that STAT1 indeed is the dominant inducer of IL-10 under strongly Th1 polarizing conditions (**Figures 6C, D**).

Given present and past evidence that STAT1 and STAT3 each promote T cell IL-10 production, we compared their distributions across relevant gene loci. To that end, we cross-referenced the STAT1 ChIP-seq dataset referenced above with a STAT3 ChIP-seq dataset that was also captured in CD4^+^ T cells treated with IL-27 (17), and a STAT4 ChIP-seq dataset captured in CD4^+^ T cells treated IL-12 (54). The latter was included because IL-12-STAT4 signaling is prevalent during acute toxoplasmosis and prior work has shown it to be important for T cell IL-10 production (55, 56). Notably, we found that STAT1, STAT3 and STAT4 co-localized at >3000 genomic regions (**Figure 6E** and **Table S5**), including multiple sites along the proximal *Il10* locus and sites associated with key IL-10 regulators, particularly MAF and IRF4 (**Figures 6E, F**, **S6** and **Table S5**). Thus, STAT1, STAT3 and STAT4 appear to compete for access to DNA regulatory elements that control expression of IL-10 and key upstream transcriptional regulators.

## Discussion

Using *Stat1 ^T-KO^* mice we demonstrate that, while not necessary to control parasite replication, T cell intrinsic STAT1 signaling is required to curb immuno-pathology during acute toxoplasmosis. This phenotype is in stark contrast to *Stat1^-/-^* mice, which are overrun by parasites and, thus, illustrate vital pro-inflammatory aspects of STAT1 (28). However, it is much like the phenotype of *Il10^-/-^* and *Il27ra^-/-^ mice*, in line with the idea that IL-27 is a major STAT1 activator which, in turn, is a major IL-10 inducer (57). Infected *Stat1 ^T-KO^* mice are further characterized by aberrant Th1 responses with diminished IL-10 and increased IL-13 production. It remains to be determined if IL-10 and/or IL-27 are also required to limit IL-13 production during acute toxoplasmosis. However, it is known that IL-27 limits IL-13 production in models of Th2-type inflammation, that it limits IL-13^+^ Th1 cells during infection with Sendai virus, and that STAT1 limits IL-13^+^ Th1 cells in settings of immunization and autoimmune disease (56–59). Here, we demonstrate that *Stat1 ^T-KO^* mice exhibit each of these properties during infection with *T. gondii* and further establish that STAT1 directly binds not only the *Il10* and *Il13* loci, but also loci encoding other transcription factors that modulate IL-10 and IL-13 production, most notably MAF and IRF4.

As with *T. gondii*, mice lacking STAT1 selectively in T cells (*Stat1* ^Lck-Cre^ mice) are able to control *Listeria monocytogenes* infection, in fact better that WT controls, but ultimately succumb to immunopathology (60). Given this parallel, it is important to consider that *Stat1 ^T-KO^* mice (and likely *Stat1* ^Lck-Cre^ mice) already bear evidence of chronic inflammation before infection. Consistent with prior work showing that pan-cellular *Stat1 ^-/-^* mice are prone to inflammatory disease (36), we find that effector/memory CD4^+^ and CD8^+^ T cells accumulate in *Stat1 ^T-KO^* mice at steady state (**Figures S1E, F**) However, CD4^+^ FOXP3^+^ Treg were also increased (**Figures S1G, H**) and baseline frequencies of IFN-γ producing CD4^+^ and CD8^+^ T cells were comparable to littermate controls (**Figure S1I**). These latter findings contrast prior studies with *Stat1 ^T-KO^* mice (30), likely due to differences in *ex vivo* re-stimulation protocols, and suggest that T cells from *Stat1 ^T-KO^* mice are not broadly hyper-inflammatory. Nevertheless, accumulation of effector/memory T cells at steady state raises the possibility that immunopathology in *Stat1 ^T-KO^* mice is not driven by excessive responses to Toxoplasma-derived antigens but, rather, from bystander activation of autoreactive cells that were already present and primed prior to infection. Further studies addressing antigen specificity of IFN-γ^+^ T cells before and after challenge would help distinguish between these non-exclusive scenarios.

STAT3 is considered both a key upstream inducer and downstream mediator of IL-10 (48). Using mice that express a dominant negative form of STAT3 (Stat3 *^HIES^*), we demonstrate that the former is not critical during acute *T. gondii* infection. Like *Stat1 ^T-KO^* mice, *Stat3 ^HIES^* mice are able to control parasite replication yet succumb to lethal immuno-pathology. However, Th1 cells from *Stat3 ^HIES^* mice do produce IL-10 and not IL-13, similar to WT controls. Given that *Stat1^T-KO HIES^* mice phenocopy *Stat1^T-KO^* mice rather than *Stat3 ^HIES^* mice, we interpret that STAT1 is dominant over STAT3 in each context. However, since the HIES transgene restricts STAT3 activity in all cells (52), we also cannot interpret findings from *Stat1^T-KO HIES^* mice solely through the lens of T cell intrinsic effects. Nevertheless, it is clear that STAT3 is not strictly required for optimal T cell IL-10 production or suppression of aberrant IL13^+^ Th1 responses during acute *T. gondii* infection.

STAT family transcription factors control gene expression *via* both direct and indirect mechanisms (46). Direct regulation involves STATs binding to DNA regulatory elements associated with a given gene, most notably promoters and enhancers, and thereby instructing transcription. Indirect regulation can take many forms. Here, we focused on the ability of STATs to induce expression other transcription factors which, in turn, modulate *Il10* and *Il13* gene expression. Building on prior studies showing STAT1 and STAT3 are each required for IL-27-driven IL-10 production *in vitro* (8), we assessed whether they directly engage gene loci for IL-10 and/or upstream regulators. Indeed, we found that STAT1 and STAT3 co-localized near *Il10* and several relevant transcription factors. Regarding the latter, MAF and IRF4 stand out because each is thought to be induced by STAT3 and necessary for optimal T cell IL-10 production (10, 47, 49). Also, unlike most of the other transcription factors that we considered, both registered as DEG in our transcriptome studies, although only MAF appeared positive regulated. Given that STAT1 and STAT3 occupy the same sites at *Il10* and other relevant gene loci, these findings argue for a ‘zero sum’ scenario whereby STAT1, STAT3 and possibly STAT4 compete for access to the same DNA regulatory elements. However, this does not preclude the possibility of cooperation. For instance, STAT1 and STAT3 could simultaneously occupy district sites within a given locus or separately control expression of other genes (i.e. transcription factors) which, in turn, cooperate at a given locus. Transcellular collaboration should also be considered; STAT1 induces T cell IL-10 production which, in turn, activates STAT3 in myeloid cells. STAT co-localization can also mediate antagonism. For instance, STAT1 and STAT3 co-localize at the *Ifng*, *Tbx21*, *Il17a* and *Rorc* loci in T cells but have opposing effects on gene transcription (17). This may also be the case at the IL-13 locus where STAT1 is clearly a negative regulator while STAT3 is clearly not. Taken together, the data presented here strengthen ties between STAT1 and STAT3 and highlight their ability to elicit IL-10 production from CD4^+^ T cells.

Beyond a lack of IL-10 production, there are other notable similarities between *Stat1 ^T-KO^* and *Il27ra^-/-^* mice. One is the paucity of T-bet-expressing Treg. Prior work has shown that STAT1 activating cytokines induce T-bet expression in Treg which, in turn, enables them to efficiently suppress Th1 type responses (9, 40, 41, 61). It is also understood that IL-27R is required for accumulation of T-bet^+^ Treg in mice challenged with *T. gondii*, and that STAT1 is required for IL-27-driven induction *in vitro* (9). Here, we connect the dots by demonstrating that STAT1 is critical *in vivo*, leading us to conclude that a lack of T-bet^+^ Treg likely contributes to the outgrowth aberrant Th1 responses in *Stat1 ^T-KO^* mice challenged with *T. gondii.* There were also notable differences between *Stat1 ^T-KO^* and *Il27ra^-/-^* mice (4). Among these is the fact that CD4^+^ T cells were clearly more impacted than CD8^+^ T cells in *Stat1 ^T-KO^*, and that IFN-γ^+^ CD4^+^ Th1 cells are not amplified. This latter finding suggests that IL-27R employs STAT1-independent mechanisms to control expansion of Th1 cells and, in turn, that STAT1 is more involved with enforcing self-regulation (i.e. IL-10 production) and suppressing unwanted IL-13 production. Given the prevalence of STAT1 stimuli in settings of acute and chronic inflammation, and the importance of IL-10 across infectious and autoimmune diseases, this finding may be relevant for a broad range clinical settings where T cell STAT1 signaling is either invoked or suppressed.

## Methods

### Animals

*Stat1* flox/flox mice were generated as described (62), backcrossed to C57Bl/6 (>8 generations), and further crossed with C57Bl/6 *Cd4* Cre mice (stain: 022071 from Jackson Labs, USA) to generate *Stat1 ^T-KO^* mice. STAT3 *^HIES^* mice were generated as described (52) and crossed *Stat1 ^T-KO^* to generate *Stat1 ^TKO HIES^* mice. *Stat1^-/^* ^-^ mice were purchased from Taconic labs (USA) and backcrossed to C57Bl/6 (>8 generations). Wild type C57BL/6J mice were purchased from Jackson Labs (Strain: 000664). For antibody quantification, serum was collected from 12–16-week-old cohorts of uninfected mice and assayed using LEGENDplex assays (service by Biolegend, USA). For histology, lungs, livers and spleens were dissected at day 7 post-infection, then processed for H&E staining. Blinded scoring was performed by a veterinary pathologist. Both male and female mice were used, and all cohorts were sex and age matched. Animals were housed and handled in accordance with NIH and University of Miami guidelines and all experiments approved by the respective Animal Care and Use Committees.

### *T. gondii* infection and quantification

Mice were injected intraperitoneally with approximately 20 cysts of the avirulent ME49 strain of *T. gondii*, as described (63). Parasite burden was determined by microscopy, specifically by counting infected cells within cytospin smears obtained from Peritoneal Exudate cells (PEx), or by PCR to detect the *T. gondii* B1 gene in DNA from whole lung, liver and spleen samples as described (64). PCR data are presented as 2^-ddCt^ comparing amplification of the B1 gene relative to the mammalian IL-2 locus such that negative values indicate that the parasite DNA is less abundant. All quantification was performed at day *7* post-infection.

### *Ex vivo* re-stimulations

Single cell suspensions were made from spleens and peritoneal exudates at day 7 post-infection as described (65). 0.5-1 x 10^6 cells/ml were then stimulated with either soluble Toxoplasma antigen (sTAg; 10 μg/ml) or plate bound anti-CD3ϵ (10 μg/ml; clone 17A2; BioXcell, USA). 18 hours later, cultures were pulsed with Brefeldin A for 2-3 hours (BFA; 10 μg/ml; Sigma, USA), then processed for intracellular flow cytometry. All cultures were in RPMI-1640 medium supplemented with 10% fetal calf serum, 1% sodium pyruvate, 1% nonessential amino acids, 10 mM HEPES, 0.1% β-Mercaptoethanol, 100 U/ml penicillin and 100 mg/ml streptomycin. For intracellular flow cytometry, cells were washed in phosphate-buffered saline supplemented with 0.5% bovine serum albumin and 0.1% sodium azide, then fixed and permeabilized with Cytofix/Cytoperm (BD Biosciences) and stained with fluorochrome-labeled anti-cytokine antibodies, in conjunction with fluorochrome labelled anti-mouse CD4, CD8, CD25, CD44, FOXP3, GATA3 and/or T-bet. Dead cells were excluded using Live/Dead aqua (Thermo Fischer). All antibodies were purchased from Thermo Fisher, BD Biosciences or Biolegend.

### *In vitro* T cell cultures

Naive CD4^+^ T cells (CD4^+^ CD44^low^ CD25^-^) were sorted from pooled lymph nodes and spleens of uninfected mice (>95% purity). 1 x 10^6 cells/ml were then stimulated with plate bound anti-CD3ϵ (10 μg/ml; clone: 17A2; BioXcell) and soluble anti-CD28 (1 μg/ml; clone;37.51 BioXcell) in the presence of IL-6, IL-12, IL-21 and/or IL-27. 72 hours later, cultures were pulsed with phorbol 12-myristate 13-acetate (PMA; 50 ng/ml; Sigma, USA) and Ionomycin (500 ng/ml; Sigma, USA) for 4 hours in concert with Brefeldin A for the final two hours (BFA; 10 μg/ml), then processed for intracellular flow cytometry. For detection of phospho-STAT proteins, cells were cultured with anti-CD3ϵ and anti-CD28 alone for 48 hours, pulsed with cytokine for 1 hour (without PMA, Ionomycin or BFA), then processed for intranuclear flow cytometry. Briefly, these were fixed with 2% formaldehyde, permeabilized with 100% methanol and stained with fluorochrome labelled anti-human/mouse pY701 STAT1 (Clone 4a; BD Biosciences) and anti-human/mouse pY703 STAT3 (Clone 4/P-STAT3; BD Biosciences) in conjunction with fluorochrome labelled anti-mouse CD4, CD25 and CD44. Mouse IL-6, IL-12, IL-21 and IL27 were purchased from R&D Systems (USA) and used at 10 ng/ml. All cultures contained anti-mouse IL-4 and IFN-γ (10 μg/ml each; clone 11B11 and XMG1.2; BioXcell).

### Transcriptome analysis

*T. gondii* infections and ex vivo sTAg re-stimulations were performed as above (without BFA), then IFN-γ capture assay performed as described (15). 0.5-2 × 10^5^ PE-labeled, IFN-γ-secreting cells were sorted per group (<90% purity) and immediate lysed and stored in Trizol regent (Thermo)(**Figure S4A**). Total RNA was later purified by phenol-chloroform extraction with GlycoBlue as co-precipitant (7-15 μg per sample; Life Technologies, USA). Poly(A)+ mRNA was then enriched by oligo-dT-based magnetic separation and single-end read libraries prepared with NEBNext Ultra RNA Library Prep Kit (New England Biolabs, USA). Sequencing was performed with a HiSeq 2500 (Illumina, USA) and 50 bp reads (20-50 × 10^6^ per sample) were aligned onto mouse genome build mm9 with *tophat2*, assembled with cufflinks and gene-level counts compiled with *htseq-count*. To minimize normalization artifacts, genes failing to reach an empirically defined count threshold were purged using *htsfilter*. 12-14 × 10^3^ genes were typically recovered post filtering, regardless of genotype or experimental condition. These were normalized and differentially expressed genes (DEG) called by quasi-likelihood F testing using edgeR (66). DEG call denotes >2 fold pairwise change and Benjamini-Hochberg (BH) adjusted p value < 0.05 (**Table S1**). Transcripts per million (TPM) were compiled with edgeR. An offset value of 1 was added to all TPM and those failing to reach a value >2 TPM in any genotype/condition were purged. *clusterProfiler* (67) was used for hypergeometric testing (HGT) of positively or negative regulated DEG against the KEGG (68) and Molecular Signatures (MSigDB) databases (**Table S2**) (69). KEGG pathway maps were rendered with *pathview*, and all other plots with ggplot2 or Datagraph (Visual Data Tools Inc., USA). All data are deposited to the NCBI Gene Expression Omnibus (GEO) under accession number :.

### ChIP-seq data mining

STAT1, STAT3 and STAT4 ChIP-seq datasets were downloaded from the NCBI Small Read Archive *via* GEO (GSE65621 and GSE22105). STAT1 and STAT3 datasets are from CD4^+^ T cells cultured *in vitro* with IL-6 or IL-27 (17). STAT4 dataset is from CD4^+^ T cells cultured *in vitro* with IL-12 (54). 50 bps reads were aligned using *bowtie* and non-redundant reads mapped to mouse genome mm9 with *macs2* with default settings and input controls as reference for peak calling (70). *homer* was used to annotate peaks and test for DNA motif enrichment. *bedtools intersect* was used for peak overlap analysis using the -wa option. Gene proximal peaks were defined as occurring within introns, exons, UTRs or <20 kb of transcriptional start or end sites. Genome browser files were rendered with IGV.

### Statistics

Statistical variances and distributions were measured by paired t test or ANOVA, as indicated. Bonferroni correction was used to account for multiple testing in RNA-seq and ChIP-seq datasets. When present, error bars denote standard deviation across >2 biological replicates.

## Data availability statement

All data and experimental protocols are available on request. All sequencing data are freely available in the NCBI GEO and SRA repositories under accession number GSE236970.

## Ethics statement

All animal studies were reviewed and approved by University of Miami, NIMAS and NIAID IACUC(s).

## Author contributions

AV, DJ and JO’S conceived of the project and supervised all studies. AV performed experiments, compiled all data and wrote the manuscript. LH contributed mice, reagents and advice. AS, DK, NV, LD and SR performed experiments. LN performed bioinformatic analysis. All authors contributed to the article and approved the submitted version.

## Supplementary material

Supplementary Figure 1 Pre-infection T cell compartments. **(A–G)** *Ex vivo* cytometry data from spleens of uninfected mice (A) Contour plots show CD3ε versus CD19 within the viable lymphocyte gate. **(B)** Box plots show total CD3ε^+^ CD19^-^ T cells or CD3ε^-^ CD19^+^ B cells. WT = Wild Type; S1 = *Stat1* ^T-KO^; H = *Stat3* ^HIES^; S1H = *Stat1* ^T-KO HIES^. **(C)** Contour plots show CD4 versus CD8α within the CD3α^+^ CD19^-^ gate. **(D)** Box plots show total CD4^+^ CD8α^-^ or CD4^-^ CD8α^+^ T cells. **(E)** Contour plots show CD44 versus CD62L within the CD4^+^ CD8α^-^ (top) or CD4^-^ CD8α^+^ gates (bottom). **(F)** Box plots show total CD44 ^high^ CD62L ^low^ effector or CD44 ^high^ CD62L ^high^ memory T cells. **(G)** Contour plots show IL-7Rα versus FOXP3 within the CD3α^+^ CD19^-^ CD4^+^ CD8α^-^ gate. **(H)** Box plots show total CD3α^+^ CD4^+^ CD8α^-^ IL-7Rα^+^ IL-2Rα^+^ Treg cells. **(I)** Flow cytometry contour plots show FOXP3 versus IFN-γ in splenic CD4^+^ (top) or CD8^+^ (bottom) T cells stimulated with anti-CD3ϵ on day 7. **(A–I)** Data are pooled from 4 experiments, each with at least 1 mouse per group. All box plots contain at least 3 biological replicates/group and points denote individual replicates. Stars denote unpaired t-test *p* values <0.05 relative to WT controls.

Supplementary Figure 2 Histological and serological characteristics. **(A)** H&E stained tissue sections from infected WT and *Stat1* ^T-KO^ mice were blindly scored for histological abnormalities. Scatter plot shows pathology scores for lung, liver and spleen at day 7. 1 = no pathology/inflammation; 2 = mild; 3 = moderate; 4 = severe. Data are pooled from 2 experiments, each with at least 2 mice per group. **(B)** Box plots show serum concentrations of the indicated cytokines at day 7 post-infection. WT = Wild Type; S1 = *Stat1* ^T-KO^; H = *Stat3* ^HIES^; S1H = *Stat1* ^T-KO HIES^. Data are pooled from 2 experiments, each with at least 2 mice per group. **(C)** Serum was collected from 20–24-week-old cohorts of uninfected mice. Scatter plot shows total IgE levels such that each point represents a biological replicate. 3 separate cohorts were examined, each with at least 2 mice per group. **(A–C)** All box plots contain at least 3 biological replicates/group and points denote individual replicates. Stars denote unpaired t-test *p* values <0.05 relative to WT controls.

Supplementary Figure 3 Post-infection T cell phenotypes. **(A-I)** Single cell suspensions were made from spleens and PEx of infected mice and re-stimulated overnight with agonist anti-CD3ε antibody. **(A)** Flow cytometry contour plots show T-bet versus IFN-γ within the CD4^-^ CD8α^+^ T cell gate at day 7. **(B)** Box plots show total CD4^-^ CD8α^+^ IFN-γ ^+^ cells. **(C)** Box plots show total IFN-γ^+^, IL-13^+^, IL-4^+^, IL-10^+^ or IL-17A^+^ CD4^+^ cells at day 7. **(D)** Flow cytometry contour plots show IL-4 versus IL-13 within the CD4^+^ CD8α^-^ T cell gate at day 7. **(E)** Box plots show percent IL-4^+^ among CD4^+^ CD8α^-^ cells. **(F)** Flow cytometry contour plots show IL-17A versus IFN-γ within the CD4^+^ CD8α^-^ T cell gate at day 7. **(G)** Box plots show percent IL-17A^+^ among CD4^+^ CD8α^-^ cells. **(H)** Flow cytometry contour plots show GATA3 versus IL-13 within the CD4^+^ CD8α^-^ T cell gate at day 7. **(A–H)** Data are pooled from 3 experiments, each with at least 2 mice per group. Stars denote unpaired t-test *p* values <0.05 relative to WT controls. All box plots contain at least 3 biological replicates/group and points denote individual replicates.

Supplementary Figure 4 STAT1 dependent genes pervade Toxoplasma-related pathways. **(A)** Single cell suspensions were made from spleens at day 7 post-infection, cultured overnight with soluble Toxoplasma antigen (sTAg), then subjected to IFN-γ capture and sorted for transcriptome analysis (see **Figures 2A**). Flow cytometry pseudocolor plots show surface staining of CD4 and IFN-γ recorded while sorting. Frequency of target CD4^+^ IFN-γ^+^ events is shown. Data are from one experiment with 2 biological replicates per group. **(B, C)** Pathview plots show STAT1-dependent DEG within the Th1 and Th2 cell differentiation (mmu04658) and toxoplasmosis (mmu05145) KEGG pathways. Color denotes transcriptional effect; Red = higher expressed in WT, Blue = higher expressed in *Stat1 ^T-KO^*. Data are from one experiment with 2 biological replicates per group.

Supplementary Figure 5 IL-10 production by conventional and regulatory T cells. **(A)** Flow cytometry contour plots show FOXP3 versus IL-10 in splenic CD4^+^ T cells stimulated with anti-CD3ϵ (day 7).

Supplementary Figure 6 Co-localization of STAT1 and STAT3 at IL-10-associated gene loci. **(A)** STAT1, STAT3 and STAT4 ChIP-seq datasets were cross-referenced. Genome browser tracks show STAT localization at gene loci associated with IL-10 production.

Supplementary Figure 7 Opposing effects of STAT1 and STAT3 on T cell differentiation. **(A)** Naive CD4^+^ T cells were purified from uninfected mice, then activated and cultured in the presence of IL-6 or IL-27 for 72 hours (control = no cytokine). Flow cytometry contour plots show IL-17A versus IFN-γ gated on viable, CD4^+^ cells. **(B)** Box plots show log2 fold change in percentage of IFN-γ^+^ IL-17A^-^ Th1 (top) and IFN-γ^-^ IL-17A^+^ Th17 cells (bottom). Data are pooled from 3 experiments, each with 1 mouse per group. Stars denote unpaired t-test *p* values <0.05 relative to WT controls. All box plots contain at least 3 biological replicates/group and points denote individual replicates.
