# Supplementary material for: T cell intrinsic STAT1 signaling prevents aberrant Th1 responses during acute toxoplasmosis
Source: Front Immunol. 2023 Jul 25;14:1212190. doi: 10.3389/fimmu.2023.1212190 (PMC10407301; doi:10.3389/fimmu.2023.1212190)
Supplement: Supplementary Figure 1 — Pre-infection T cell compartments. (A–G) Ex vivo cytometry data from spleens of uninfected mice (A) Contour plots show CD3ε versus CD19 within the viable lymphocyte gate. (B) Box plots show total CD3ε+ CD19- T cells or CD3ε- CD19+ B cells. WT = Wild Type; S1 = Stat1 T-KO; H = Stat3 HIES; S1H = Stat1 T-KO HIES. (C) Contour plots show CD4 versus CD8α within the CD3α+ CD19- gate. (D) Box plots show total CD4+ CD8α- or CD4- CD8α+ T cells. (E) Contour plots show CD44 versus CD62L within the CD4+ CD8α- (top) or CD4- CD8α+ gates (bottom). (F) Box plots show total CD44 high CD62L low effector or CD44 high CD62L high memory T cells. (G) Contour plots show IL-7Rα versus FOXP3 within the CD3α+ CD19- CD4+ CD8α- gate. (H) Box plots show total CD3α+ CD4+ CD8α- IL-7Rα+ IL-2Rα+ Treg cells. (I) Flow cytometry contour plots show FOXP3 versus IFN-γ in splenic CD4+ (top) or CD8+ (bottom) T cells stimulated with anti-CD3ϵ on day 7. (A–I) Data are pooled from 4 experiments, each with at least 1 mouse per group. All box plots contain at least 3 biological replicates/group and points denote individual replicates. Stars denote unpaired t-test p values <0.05 relative to WT controls. [file DataSheet_1.pdf]

T cell intrinsic STAT1 signaling prevents aberrant Th1 responses during acute toxoplasmosis

Authors: Aaron B. Schultz [1,2], David G. Kugler [7], Luis Nivelo [1,2], Nicolas Vitari [1,2,3], Laura P. Doyle [2,4], Svetlana Ristin [1,2], Lothar Hennighausen [5], John J. O'Shea [6], Dragana Jankovic [7] † and Alejandro V. Villarino [1,2] † *

Affiliations:

[1] Department of Microbiology and Immunology, Miller School of Medicine, University of Miami, Miami, FL, USA

[2] Sylvester Comprehensive Cancer Center, University of Miami, Miami, USA

[3] Department of Surgery, Miller School of Medicine, University of Miami, Miami, FL, USA

[4] Department of Molecular and Cellular Pharmacology, Miller School of Medicine, University of Miami, Miami, FL, USA

[5] National Institute of Diabetes, Digestive and Kidney Diseases, National Institutes of Health, Bethesda, MD, USA

[6] Lymphocyte Cell Biology Section, Molecular Immunology and Inflammation Branch, National Institute of Arthritis, Musculoskeletal and Skin Diseases, National Institutes of Health, Bethesda, MD, USA

[7] Immunoparasitology Unit, Laboratory of Parasitic Diseases, National Institute of Allergy and Infectious Diseases, National Institutes of Health, Bethesda, MD, USA.

* Corresponding author: Alejandro V. Villarino (alejandro.villarino@miami.edu)

† These authors share senior authorship

Abstract: Infection-induced T cell responses must be properly tempered and terminated to prevent immuno-pathology. Using transgenic mice, we demonstrate that T cell intrinsic STAT1 signaling is required to curb inflammation during acute infection with *Toxoplasma gondii*. Specifically, we report that mice lacking STAT1 selectively in T cells expel parasites but ultimately succumb to lethal immuno-pathology characterized by aberrant Th1-type responses with reduced IL-10 and increased IL-13 production. We also find that, unlike STAT1, STAT3 is not required for induction of IL-10 or suppression of IL-13 during acute toxoplasmosis. Each of these findings was confirmed *in vitro* and ChIP-seq data mining showed that STAT1 and STAT3 co-localize at the *Il10* locus, as well as loci encoding other transcription factors that regulate IL-10 production, most notably *Maf* and *Irf4*. These data advance basic understanding of how infection-induced T cell responses are managed to prevent immuno-pathology and provide specific insights on the anti-inflammatory properties of STAT1, highlighting its role in shaping the character of Th1-type responses.

Summary: T cell STAT1 signaling limits immuno-pathology

Supplemental Figure Legends

Figure S1. *Pre-infection T cell compartments.* (A-G) *Ex vivo* cytometry data from spleens of uninfected mice (A) Contour plots show CD3ε versus CD19 within the viable lymphocyte gate. (B) Box plots show total CD3ε$^+$ CD19$^-$ T cells or CD3ε$^-$ CD19$^+$ B cells. WT = Wild Type; S1 = *Stat1* $^{\text{T-KO}}$; H = *Stat3* ^{HIES}; S1H = *Stat1* $^{\text{T-KO HIES}}$. (C) Contour plots show CD4 versus CD8α within the CD3ε$^+$ CD19$^-$ gate. (D) Box plots show total CD4$^+$ CD8α$^-$ or CD4$^-$ CD8α$^+$ T cells. (E) Contour plots show CD44 versus CD62L within the CD4$^+$ CD8α$^-$ (top) or CD4$^-$ CD8α$^+$ gates (bottom). (F) Box plots show total CD44 ^{high} CD62L ^{low} effector or CD44 ^{high} CD62L ^{high} memory T cells. (G) Contour plots show IL-7Rα versus FOXP3 within the CD3ε$^+$ CD19$^-$ CD4$^+$ CD8α$^-$ gate. (H) Box plots show total CD3ε$^+$ CD4$^+$ CD8α$^-$ IL-7Rα$^+$ IL-2Rα$^+$ Treg cells. (I) Flow cytometry contour plots show FOXP3 versus IFN-γ in splenic CD4$^+$ (top) or CD8$^+$ (bottom) T cells stimulated with anti-CD3ε on day 7. (A-I) Data are pooled from 4 experiments, each with at least 1 mouse per group. All box plots contain at least 3 biological replicates/group and points denote individual replicates. Stars denote unpaired t-test *p* values <0.05 relative to WT controls.

Figure S2. *Histological and serological characteristics.* (A) H&E stained tissue sections from infected WT and *Stat1* $^{\text{T-KO}}$ mice were blindly scored for histological abnormalities. Scatter plot shows pathology scores for lung, liver and spleen at day 7. 1 = minimal; 2 = mild; 3 = moderate; 4 = severe. For all tissues, 'minimal' is defined as isolated patches of inflammation similar to background lesions seen in conventionally housed control mice. For lungs, 'severe' is defined as confluent inflammation with disruption of normal architecture. Lung inflammation was typically centered around pulmonary arteries (perivasculitis and vasculitis) For livers, 'severe' is defined as entire lobules being effaced by inflammation. Milliary histiocytic hepatitis, capsulitis and perivasculitis were the typical pathologies. For spleen, severe is defined as greater than 75% of the splenic section is involved and/or prominent patches of necrosis. Both red and while pulp were affected. White pulp inflammation was typically centered on periarteriolar lymphocyte sheaths with sparing of B-cell areas. Data are pooled from 2 experiments, each with at least 2 mice per group. (B) Box plot show serum concentrations of the indicated cytokines at day 7 post-infection. WT = Wild Type; S1 = *Stat1* $^{\text{T-KO}}$; H = *Stat3* ^{HIES}; S1H = *Stat1* $^{\text{T-KO HIES}}$. Data are pooled from 2 experiments, each with at least 2 mice per group. (C) Serum was collected from 20-24 week old cohorts of uninfected mice. Scatter plot shows total IgE levels such that each point represents a biological replicate. 3 separate cohorts were examined, each with at least 2 mice per group. (A-C) All box plots contain at least 3 biological replicates/group and points denote individual replicates. Stars denote unpaired t-test *p* values <0.05 relative to WT controls.

Figure S3. *Post-infection T cell phenotypes.* (A-I) Single cell suspensions were made from spleens and PEx of infected mice infected and re-stimulated overnight with agonist anti-CD3ε antibody. (A) Flow cytometry contour plots show T-bet versus IFN-γ within the CD4$^-$ CD8α$^+$ T cell gate at day 7. (B) Box plots show total CD4$^-$ CD8α$^+$ IFN-γ^+ cells. (C) Box plots show total IFN-γ^+, IL-13$^+$, IL-4$^+$, IL-10$^+$ or IL-17A$^+$ CD4$^+$ cells at day 7. (D) Flow cytometry contour plots show IL-4 versus IL-13 within the CD4$^+$ CD8α$^-$ T cell gate at day 7. (E) Box plots show percent IL-4$^+$ among CD4$^+$ CD8α$^-$ cells. (F) Flow cytometry contour plots show IL-17A versus IFN-γ within the CD4$^+$ CD8α$^-$ T cell gate at day 7. (G) Box plots show percent IL-17A$^+$ among CD4$^+$ CD8α$^-$ cells. (H) Flow cytometry contour plots show GATA3 versus IL-13 within the CD4$^+$ CD8α$^-$

T cell gate at day 7. (A-H) Data are pooled from 3 experiments, each with at least 2 mice per group. Stars denote unpaired t-test *p* values <0.05 relative to WT controls. All box plots contain at least 3 biological replicates/group and points denote individual replicates.

Figure S4. *STAT1 dependent genes pervade Toxoplasma-related pathways.* (A) Single cell suspensions were made from spleens at day 7 post-infection, cultured overnight with soluble Toxoplasma antigen (sTAg), then subjected to IFN-γ capture and sorted for transcriptome analysis (see Fig. 2A). Flow cytometry pseudocolor plots show surface staining of CD4 and IFN-γ recorded while sorting. Frequency of target $CD4^+$ $IFN\text{-}\gamma^+$ events is shown. Data are from one experiment with 2 biological replicates per group. (B-C) Pathview plots show STAT1-dependent DEG (per Fig. 2B) within the Th1 and Th2 cell differentiation (mmu04658) and toxoplasmosis (mmu05145) KEGG pathways. Color denotes transcriptional effect; Red = higher expressed in WT, Blue = higher expressed in $Stat1^{T\text{-}KO}$. Data are from one experiment with 2 biological replicates per group.

Figure S5. *IL-10 production by conventional and regulatory T cells.* (A) Flow cytometry contour plots show FOXP3 versus IL-10 in splenic $CD4^+$ T cells stimulated with anti-CD3ε (day 7).

Figure S6. *Co-localization of STAT1 and STAT3 at IL-10-associated gene loci*. (A) STAT1, STAT3 and STAT4 ChIP-seq datasets were cross-referenced. Genome browser tracks show STAT localization at gene loci associated with IL-10 production.

Figure S7. *Opposing effects of STAT1 and STAT3 on T cell differentiation.* (A) Naive $CD4^+$ T cells were purified from uninfected mice, then activated and cultured in the presence of IL-6 or IL-27 for 72 hours (control = no cytokine). Flow cytometry contour plots show IL-17A versus IFN-γ gated on viable, $CD4^+$ cells. (B) Box plots show log2 fold change in percentage of $IFN\text{-}\gamma^+$ $IL\text{-}17A^-$ Th1 (top) and $IFN\text{-}\gamma^-$ $IL\text{-}17A^+$ Th17 cells (bottom). Data are pooled from 3 experiments, each with 1 mouse per group. Stars denote unpaired t-test *p* values <0.05 relative to WT controls. All box plots contain at least 3 biological replicates/group and points denote individual replicates.

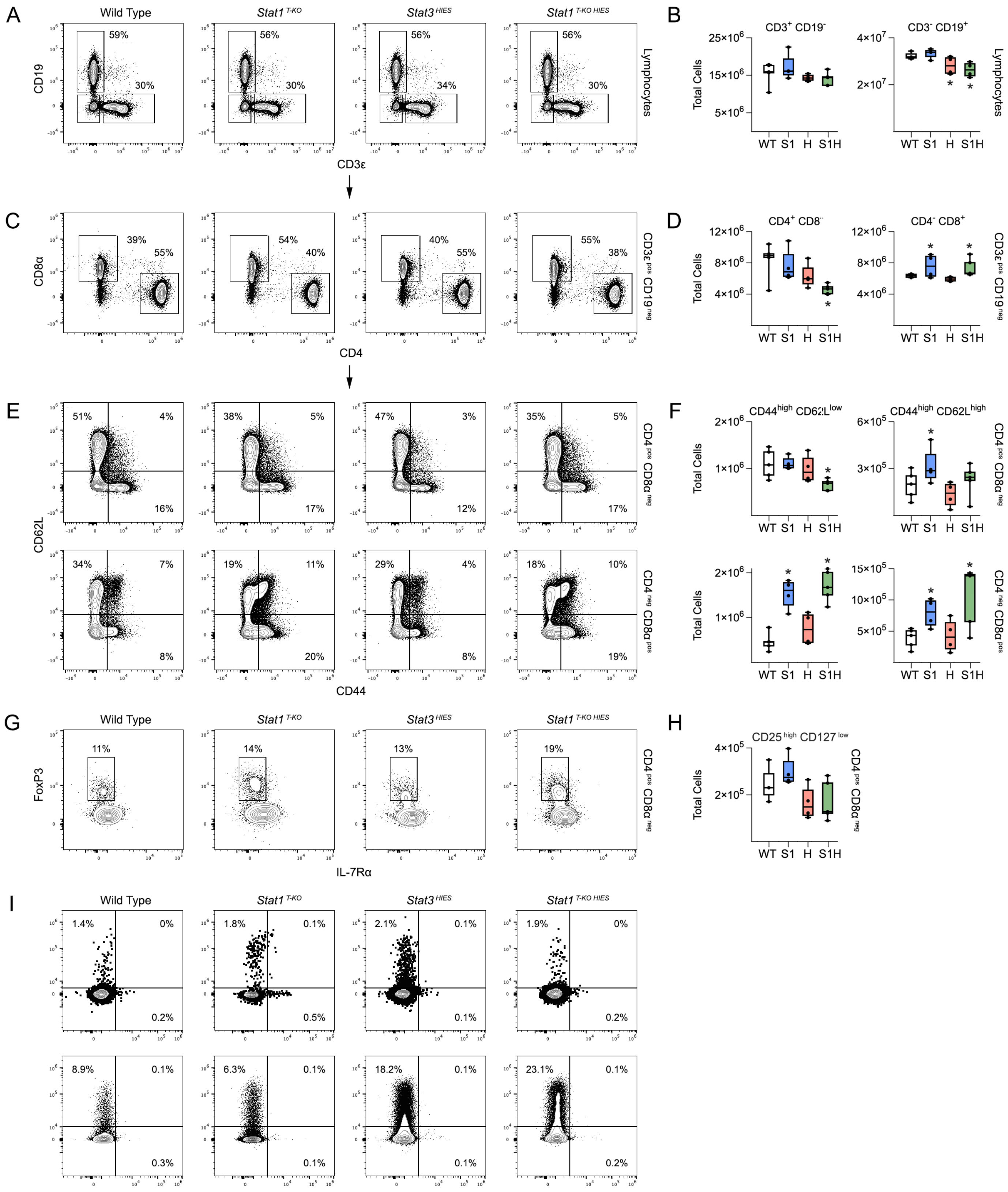
A
Wild Type
Stat1 T-KO
Stat3 HIES
Stat1 T-KO HIES
CD19
CD3ε
Lymphocytes
B
CD3+ CD19-
CD3- CD19+
Total Cells
WT S1 H S1H
C
CD8α
CD4
CD3ε pos CD19 neg
D
CD4+ CD8-
CD4- CD8+
E
CD62L
CD44
CD4 pos CD8α neg
CD4 neg CD8α pos
F
CD44high CD62Llow
CD44high CD62Lhigh
G
FoxP3
IL-7Rα
H
CD25 high CD127 low
I

Figure S1

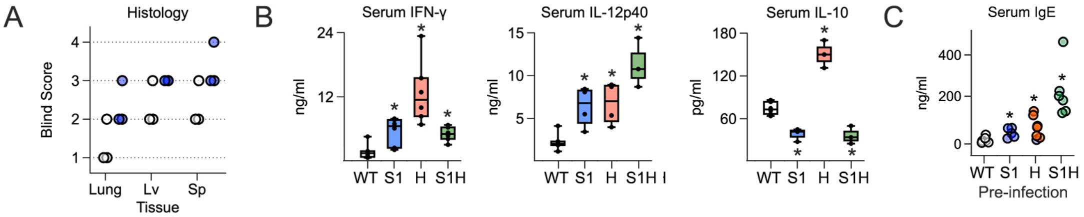
A
Histology
Blind Score
4
3
2
1
Lung
Lv
Sp
Tissue
B
Serum IFN-γ
ng/ml
24
12
WT
S1
H
S1H
Serum IL-12p40
ng/ml
15
10
5
WT
S1
H
S1H
Serum IL-10
pg/ml
180
120
60
WT
S1
H
S1H
C
Serum IgE
ng/ml
400
200
0
WT
S1
H
S1H
Pre-infection

Figure S2

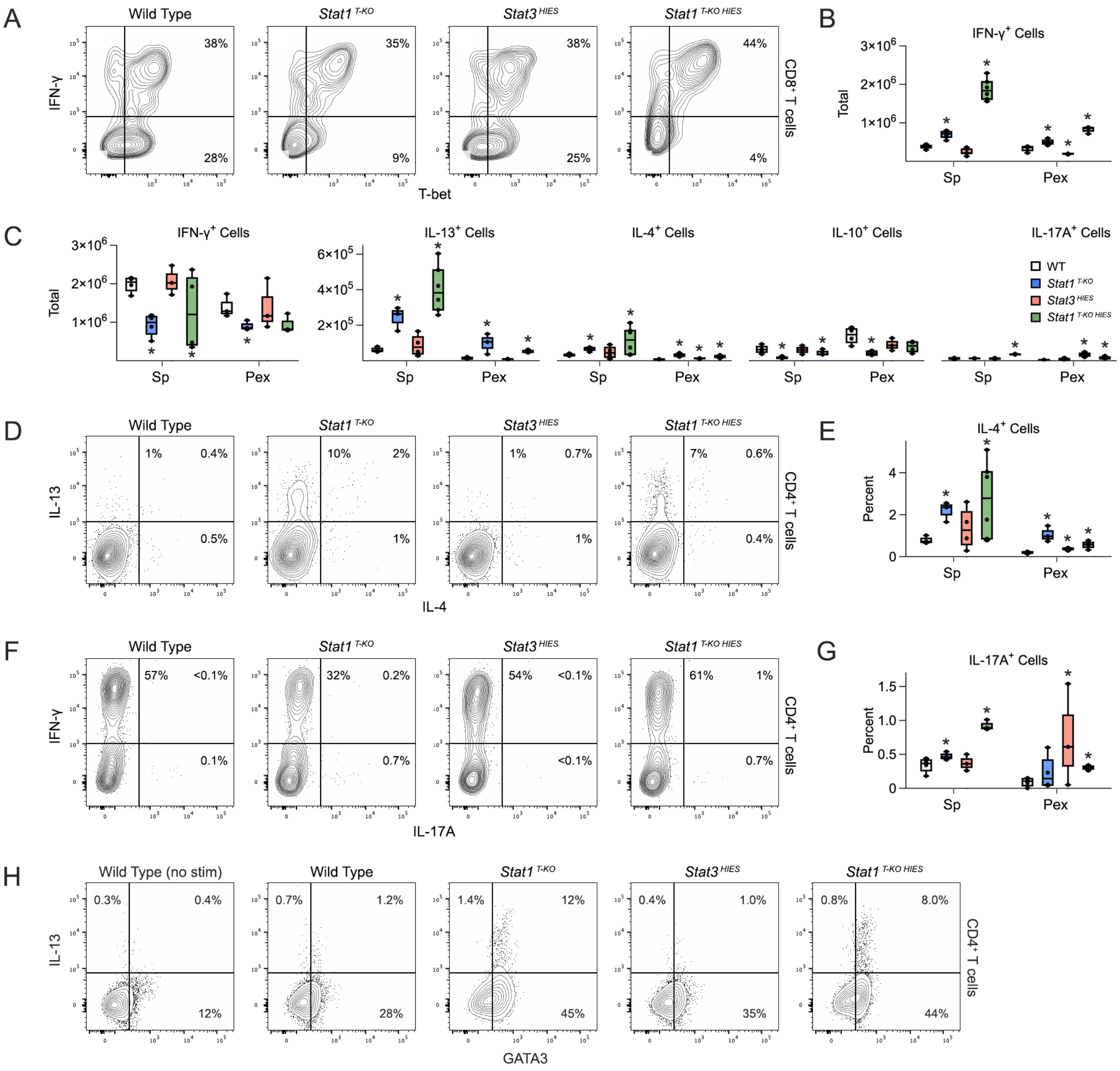
A
Wild Type
Stat1 T-KO
Stat3 HIES
Stat1 T-KO HIES
IFN-γ
T-bet
CD8+ T cells
38%
28%
35%
9%
38%
25%
44%
4%
B
IFN-γ+ Cells
Total
Sp
Pex
C
IFN-γ+ Cells
IL-13+ Cells
IL-4+ Cells
IL-10+ Cells
IL-17A+ Cells
WT
Stat1 T-KO
Stat3 HIES
Stat1 T-KO HIES
D
IL-13
IL-4
CD4+ T cells
1%
0.4%
0.5%
10%
2%
1%
1%
0.7%
1%
7%
0.6%
0.4%
E
IL-4+ Cells
Percent
F
IL-17A
57%
<0.1%
0.1%
32%
0.2%
0.7%
54%
<0.1%
<0.1%
61%
1%
0.7%
G
IL-17A+ Cells
H
Wild Type (no stim)
GATA3
0.3%
0.4%
12%
0.7%
1.2%
28%
1.4%
12%
45%
0.4%
1.0%
35%
0.8%
8.0%
44%

Figure S3

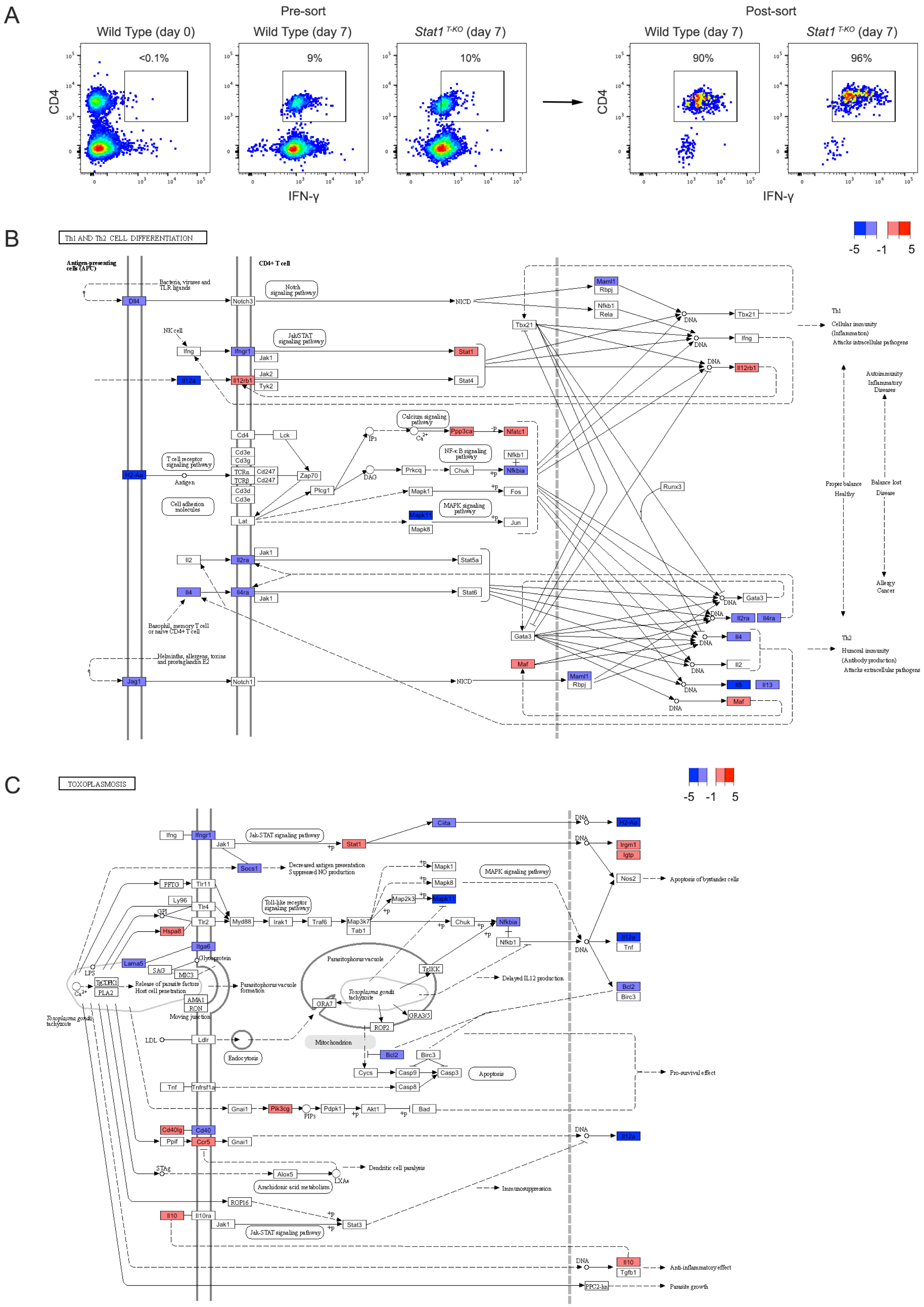
A
Pre-sort
Post-sort
Wild Type (day 0)
Wild Type (day 7)
Stat1 T-KO (day 7)
Wild Type (day 7)
Stat1 T-KO (day 7)
<0.1%
9%
10%
90%
96%
CD4
IFN-γ
B
Th1 AND Th2 CELL DIFFERENTIATION
-5
-1
5
Antigen-presenting cells (APC)
CD4+ T cell
Th1
Cellular immunity
(Inflammation)
Attacks intracellular pathogens
Autoimmunity
Inflammatory
Diseases
Proper balance
Healthy
Balance lost
Disease
Allergy
Cancer
Th2
Humoral immunity
(Antibody production)
Attacks extracellular pathogens
C
TOXOPLASMOSIS
-5
-1
5
Apoptosis of bystander cells
Delayed IL12 production
Pro-survival effect
Dendritic cell paralysis
Immunosuppression
Anti-inflammatory effect
Parasite growth

Figure S4

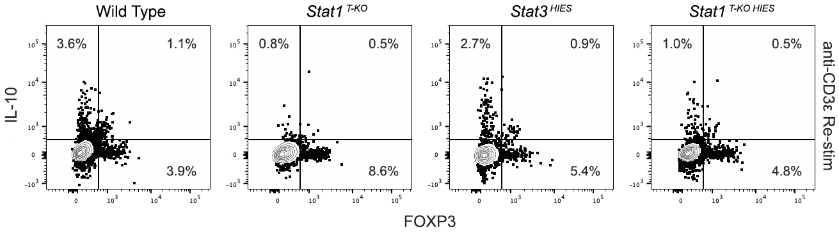
Wild Type
Stat1 T-KO
Stat3 HIES
Stat1 T-KO HIES
3.6%
1.1%
3.9%
0.8%
0.5%
8.6%
2.7%
0.9%
5.4%
1.0%
0.5%
4.8%
IL-10
FOXP3
anti-CD3ε Re-stim

Figure S5

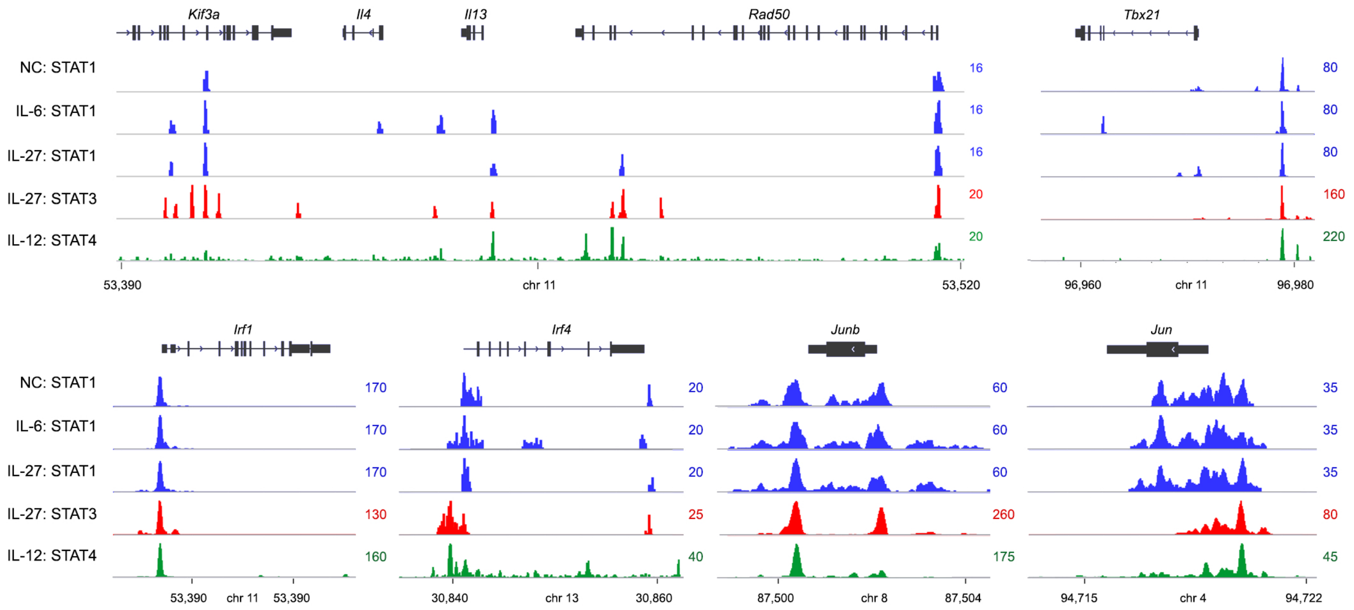
Kif3a
Il4
Il13
Rad50
Tbx21
NC: STAT1
IL-6: STAT1
IL-27: STAT1
IL-27: STAT3
IL-12: STAT4
16
16
16
20
20
80
80
80
160
220
53,390
chr 11
53,520
96,960
chr 11
96,980
Irf1
Irf4
Junb
Jun
170
170
170
130
160
20
20
20
25
40
60
60
60
260
175
35
35
35
80
45
53,390
chr 11
53,390
30,840
chr 13
30,860
87,500
chr 8
87,504
94,715
chr 4
94,722

Figure S6

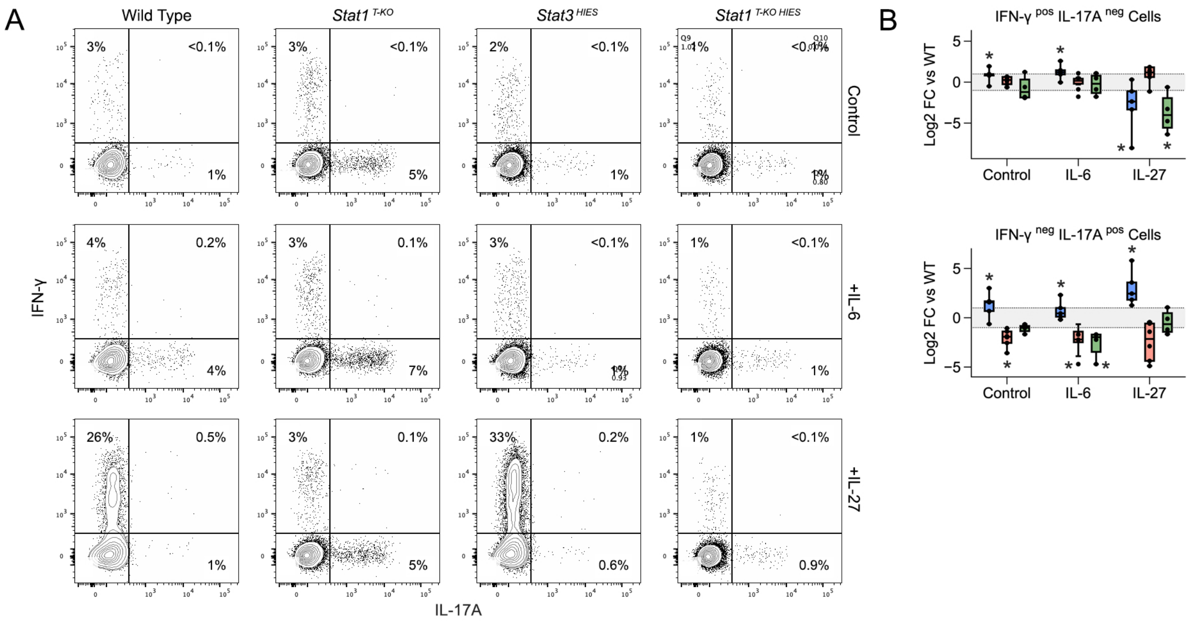
A
Wild Type
Stat1 T-KO
Stat3 HIES
Stat1 T-KO HIES
Control
+IL-6
+IL-27
IFN-γ
IL-17A
3%
<0.1%
1%
3%
<0.1%
5%
2%
<0.1%
1%
1%
<0.1%
1%
4%
0.2%
4%
3%
0.1%
7%
3%
<0.1%
1%
1%
<0.1%
1%
26%
0.5%
1%
3%
0.1%
5%
33%
0.2%
0.6%
1%
<0.1%
0.9%
B
IFN-γ pos IL-17A neg Cells
IFN-γ neg IL-17A pos Cells
Log2 FC vs WT
Control
IL-6
IL-27

Figure S7
